# Omalizumab therapy in three patients with chronic autoimmune urticaria

**DOI:** 10.4103/0256-4947.70567

**Published:** 2010

**Authors:** Mona Al-Ahmad

**Affiliations:** From the Department of Allergy, Al-Rashed Allergy Center, Kuwait

## Abstract

Chronic urticaria is a common skin disease. In about 45% of patients the cause is an autoantibody directed at the α-subunit of the high-affinity IgE receptor (chronic autoimmune urticaria; CAU). Omalizumab is a monoclonal anti-IgE antibody that has a proven role in the treatment of various allergic diseases. We gave omalizumab once every month for 16 weeks to three patients that were refractory to standard treatment, including high doses of antihistamines, leukotriene receptor antagonist, and corticosteroid. There was dramatic improvement in the primary efficacy variable—the change in mean urticaria activity score (UAS) from baseline (i.e., the average over the first 4-week period before omalizumab) to the final 4-week period of omalizumab treatment. There was improvement in the secondary efficacy variables, which included change from baseline in interference with sleep, interference with daily activities, daily diary record of urticaria signs and symptoms based on a scoring system, and rescue medication use. These improvements persisted for 12 weeks after discontinuation of the drug. Omalizumab may have a role in treating refractory cases of CAU.

Urticaria is a common skin disorder that may impair quality of life. Chronic urticaria is defined as the daily or almost daily occurrence of hives for at least 6 weeks. Chronic autoimmune urticaria (CAU) can be found in 45% of such cases, with antibodies against the high-affinity IgE receptor (IgEfcR) or IgE.^1^,^2^ The causal role of IgE in allergic disease is well established.^3^ The allergic cascade is initiated when IgE bound to high-affinity FceRI receptors on the surface of basophils and mast cells is cross-linked by allergen, resulting in degranulation of effector cells and release of histamine and leukotrienes. Omalizumab (Xolair) is a recombinant humanized monoclonal anti-IgE antibody approved for the treatment of moderate to severe persistent asthma; it acts by binding to the C3 domain of the heavy chain of IgE.^4^ It interrupts the allergic cascade by forming complexes with IgE and down-regulating FceRI as a direct consequence of the reduction in free IgE concentration. By preventing IgE binding to IgEfcR, omalizumab improves patient symptoms and may thus be a novel therapy for CAU.^5^

Treatment of CAU can be challenging for both patients and physicians. Some CAU patients may have a partial or unsatisfactory response to standard therapy, including high doses of antihistamines, leukotriene receptor antagonist, and corticosteroid. We present three cases of CAU that were refractory to standard therapy. All patients received omalizumab for 16 weeks.

## CASES

Three patients, aged 24, 39, and 49 years (all females), were diagnosed with CAU. They all had recurrent attacks of urticaria and angioedema affecting the whole body. The urticarial lesions lasted for less than 24 hours. They had no evidence of physical urticaria, urticarial vasculitis, or urticaria secondary to any underlying disease, and there was no history of bronchial asthma, allergic rhinitis, or any other atopic disease. Physical exam was normal and there was no evidence of dermographism. Baseline characteristics and test results are given in **Table 1**. All patients had a positive autologous skin test to serum. This test involves the intradermal injection of 0.05 mL of both sterile autologous serum (ASST) and plasma (APST), with intradermal saline as a negative control, and looking for wheal formation. Serum and plasma samples were centrifuged at 2500 rpm for 5 min and immediately used for the intradermal tests. Readings were taken at 30 minutes. Only an unequivocal wheal-and-flare reaction, with a wheal diameter of at least 1.5 mm more than control, was taken as a positive test result.^6^,^7^ All patients showed a less-than-satisfactory response to maximal doses of antihistamine therapy (hydroxyzine two 25-mg tablets every 6 hours, levocetrizine two 5-mg tablets a day, ranitidine 150 mg twice a day, and montelukast 10 mg orally taken once daily on most days prior to omalizumab therapy. They were kept symptom free on a daily oral corticosteroid for a duration of 2 to 3 months. They refused other steroid-sparing agents such as cyclosporine, methotrexate, or other immunosuppressant medications for fear of side effects. They were gradually weaned off all medications, except the antihistamine, 4 weeks before the start of omalizumab. They were taking 25 to 50 mg of hydroxyzine as often as 4 times a day as needed. After signing an informed consent form, they received omalizumab for 16 weeks. Omalizumab was dosed according to body weight. Serum IgE was obtained 4 weeks before omalizumab treatment was started. Each patient received 300 mg of omalizumab subcutaneously every 4 weeks. All patients maintained a daily diary to record urticaria signs and symptoms based on a scoring system (0-3). Pruritus severity had to be at least moderate (score of 2 on a 0-3 scale), and the urticaria activity score (UAS) **(Table 2)**—a combination of pruritus severity, number of hives, and size of largest hive–had to be at least moderate (minimum score of 4 on a 0-9 scale). At monthly visits, the mean UAS was calculated and rescue medication use was recorded. Mean (standard deviation) UAS declined significantly from baseline to the final 4-week period of omalizumab treatment (8.52 [0.51] to 0.23 [0.42]; *P*<.01) for patient 1 and by a similar degree for the other two patients (**Table 3**). All patients showed complete resolution of symptoms. There was a significant change from baseline in mean score for interference with sleep (from 1.71 (0.46) to 0.00) with *P*<.01 for patient 1, from 2.48 (0.72) at baseline to 0.00 (*P*<.01) for patient 2, and from 2.48 (0.63) at baseline to 0.00 (*P*<.01) for patient 3. All patients had significant improvement from baseline in interference with daily activities, with a decrease in the mean score from baseline in interference with daily activity 2.61 (0.49) to 0.00 (*P*<.01) for patient 1, from 2.61 (0.49) to 0.00 for (*P*<.01) for patient 2, and from 2.48 (0.51) at base line to 0.03 (0.018) (*P*<.01) for patient 3. Treatment with omalizumab resulted in a significant decrease in mean rescue medication use for all patients. The decrease in mean rescue medication for patient 1 was from 4.00 tablets daily at baseline to 0.10 (0.3) tablets during the final 4-week period of omalizumab treatment, from 5.84 (0.37) tablets at baseline to 0.32 (0.54) tablets for patient 2, and from 5.48 (0.51) tablets to 0.39 (0.56) tablets for patient 3. The decrease in mean rescue use of hydroxyzine was significant (*P*<.01) for all patients. They all were followed up for 12 weeks after they had discontinued omalizumab, and there were no reports of symptom recurrence or of other adverse effects.

**Table 1:** Patient characteristics

	Patient 1	Patient 2	Patient 3
Age (years)	24	39	49
Sex	F	F	F
Duration of symptoms (years) (continuous)	4	4	5
Dermographism	Negative	Negative	Negative
Total serum IgE (IU/mL)	492.89	300.57	463.72
Anti-thyroglobulin antibody (IU/mL)	55	(+) (343)	2
Anti-microsomal antibody (IU/mL)	(+) (70)	(+) (806)	(+) (55)
Anti-*Helicobacter pylori* antibody (U/mL)	(+) (135)	(+) (280)	(+) (310)
Autologous skin test	(+) to serum	(+) to serum	(+) to serum

(+) positive, titer shown in parentheses. Autoantibody titers were before the start of the therapy with omalizumab

**Table 2:** Scoring system for urticaria severity

Score	Pruritis sevirity	Number of hives	Size of largest hive (cm)	Interference with sleep	Interference with daily activities	Erythema severity
0	None	None	None	None	None	None
1	Mild, minimal awareness, easily tolerated	1 to 6	<1.25	Mild, not troublesome, adequate sleep	Mild, not troublesome, little effect on activity	Slight
2	Moderate, definite awareness, bothersome but tolerable	7 to 12	1.25-2.5	Moderate, awoke occasionally, average sleep	Moderate, some interference with activity	Moderate
3	Severe, difficult to tolerate	>12	>2.5	Severe, substantial interference with sleep, poor sleep	Severe, daily activities substantially or completely curtailed	Significant

**Table 3:** Urticaria activity score (mean) for the three patients at different times after receiving omalizumab.

Week of omalizumab	Patient 1	Patient 2	Patient 3
Week 0	8.52	8.48	7.61
Week 4	4.03	4.26	5.52
Week 8	2.16	2.19	3.35
Week 12	0.61	1.87	1.65
Week 16	0.23	0.26	1.61

*P*<.05 for comparison of week-to-week scores

## DISCUSSION

We reasoned that omalizumab, by decreasing circulating IgE levels, would secondarily decrease the IgE receptor density on basophils and cutaneous mast cells and thus prevent activation by autoantibodies. In addition, omalizumab has been reported to ameliorate the symptoms of chronic urticaria in an uncontrolled study of three patients, two of whom also had IgE-mediated asthma.^8^–^11^ An explanation for these observations could be decreased expression of surface IgEfcR due to omalizumab administration.^12^,^13^ In our patients, the mean UAS decreased, the mean rescue medication use declined, and overall therapeutic response and quality of life improved after omalizumab use. This consistency in effect across our three patients, along with outcome measures, suggests that omalizumab may be useful in the treatment of patients with CAU not responsive to antihistamines or other classical therapy. Although none of our patients had asthma, the dose and dosing frequency were based on the total serum IgE and body weight, as is done in asthma patients otherwise eligible for omalizumab therapy. A recent case report suggested that response of CAU to omalizumab therapy was associated with markedly decreased levels of serum donor basophil activation as compared to patients not using omalizumab.^10^ Similarly, there are many reports supporting use of omalizumab as an effective therapy for relieving and controlling symptoms of patients with CAU. Kaplan et al^11^ recently reported on the beneficial effect of omalizumab in 12 patients with CAU. IgG autoantibodies to the a-subunit of the high-affinity IgE receptor are the cause of the urticaria in many patients with CAU. Kaplan et al proposed that omalizumab may downregulate FceRI expression, preventing mast cell activation by IgG autoantibodies. However, there are reports to the contrary, with patients getting worse with omalizumab therapy. A patient with a dual diagnosis of oral corticosteroid-dependent asthma and CAU received omalizumab for her asthma. Although asthma symptoms improved, this patient, and others, had paradoxical worsening of urticaria immediately after injection of omalizumab.^14^,^15^ Apparently, the presence of detectable autoantibodies to IgEfCR is not required for response to omalizumab, and successful therapy is not invariably associated with decrease in the levels of these autoantibodies when they are present.^8^ Patients with CAU can have negative test results for basophil activation and negative results on autologous skin testing.^16^

CAU involves a number of mechanisms in addition to IgEfCR autoantibodies. For example, potential interactions with thyroid autoimmunity and the coagulation pathway may alter mast cell activation.^17^ There is a high prevalence of thyroid autoantibodies in patients with CAU.^18^ In our series, all patients had high titers of anti-microsomal antibodies, and one had positive anti-thyroglobulin antibody, a finding consistent with the high prevalence of these thyroid antibodies.

The interpretation of the results might have been different had the sample size been bigger or if we had tried to assess long-term prognosis by increasing the period off-treatment to more than 12 weeks. There is no data available at present on the long-term effect of omalizumab in patients with urticaria. Although our experience with omalizumab is limited by the small number of patients, the outcome measures were primarily patient-reported and covered all aspects of subjective assessment of CUA measures.

To summarize, we present three patients with CAU who were refractory to standard treatment but showed good response to treatment with omalizumab. We believe that omalizumab has a role in some patients with CAU and may be useful for treatment of cases not responsive to classical therapy. Further studies should be performed to confirm these findings.
